# Identification of ovarian cancer subtype-specific network modules and candidate drivers through an integrative genomics approach

**DOI:** 10.18632/oncotarget.6774

**Published:** 2015-12-28

**Authors:** Di Zhang, Peng Chen, Chun-Hou Zheng, Junfeng Xia

**Affiliations:** ^1^ Institute of Health Sciences, School of Computer Science and Technology, Anhui University, Hefei, Anhui 230601, China; ^2^ Co-Innovation Center for Information Supply and Assurance Technology, Anhui University, Hefei, Anhui 230601, China; ^3^ College of Electrical Engineering and Automation, Anhui University, Hefei, Anhui 230601, China

**Keywords:** ovarian cancer, cancer subtype, network module, driver gene, integrative genomics approach

## Abstract

Identification of cancer subtypes and associated molecular drivers is critically important for understanding tumor heterogeneity and seeking effective clinical treatment. In this study, we introduced a simple but efficient multistep procedure to define ovarian cancer types and identify core networks/pathways and driver genes for each subtype by integrating multiple data sources, including mRNA expression, microRNA expression, copy number variation, and protein-protein interaction data. Applying similarity network fusion approach to a patient cohort with 379 ovarian cancer samples, we found two distinct integrated cancer subtypes with different survival profiles. For each ovarian cancer subtype, we explored the candidate oncogenic processes and driver genes by using a network-based approach. Our analysis revealed that alterations in DLST module involved in metabolism pathway and NDRG1 module were common between the two subtypes. However, alterations in the RB signaling pathway drove distinct molecular and clinical phenotypes in different ovarian cancer subtypes. This study provides a computational framework to harness the full potential of large-scale genomic data for discovering ovarian cancer subtype-specific network modules and candidate drivers. The framework may also be used to identify new therapeutic targets in a subset of ovarian cancers, for which limited therapeutic opportunities currently exist.

## INTRODUCTION

Ovarian cancer is a major cause of cancer-related mortality in women, with an estimated 21,290 new cases and 14,180 deaths predicted for 2015 in the United States [1]. Over the past few decades, genetic studies have elucidated some crucial genetic alterations implicated in the pathogenesis of ovarian cancer. The rapid development of next-generation sequencing technologies in recent years has facilitated the identification of numerous somatic genetic alterations in ovarian cancer. These somatic genetic alterations are classified as drivers or passengers, and distinguishing these two remains a challenge in cancer research.

Instead of individual genes, signaling pathways and networks control the biology of tumorigenesis and cancer development. Expert-curated pathways have been employed to interpret genetic alterations [2, 3]. Although helpful, these approaches are restricted by the coverage of curated pathways [4]. Consequently, network-based methods such as NetWalker [5] and Netbox [6] have been developed and extensively used to extract the subnetworks that are enriched with genetic alterations.

Network-based approaches elucidate the system level of complex genetic alterations. However, current studies often compare all tumor samples with normal samples, thereby identifying signaling pathways common to all cancer samples but ignoring the heterogeneity. Tumor subtypes represent different biological processes; thus, cancer subtype analysis is suitable to understand the cancer heterogeneity and seek therapy treatment for different subtypes. Various subtypes of ovarian cancer have been recently identified from the different types of data and methods used. For example, The Cancer Genome Atlas (TCGA) identified four transcriptional subtypes based on gene expression data [2]. Tothill et al. [7] applied an unsupervised clustering to the mRNA data of epithelial ovarian cancer and identified six subtypes. Yuan et al. [8] identified three subtypes derived from ovarian cancer microRNA (miRNA) expression through non-negative matrix factorization. However, investigation of tumor subtypes based on the combination of genetic and epigenetic factors was often ignored. Moreover, subtype and network analyses play vital roles in cancer research, but existing studies usually performed subtype analysis in isolation and failed to determine the driving force behind each subtype.

**Figure 1:**
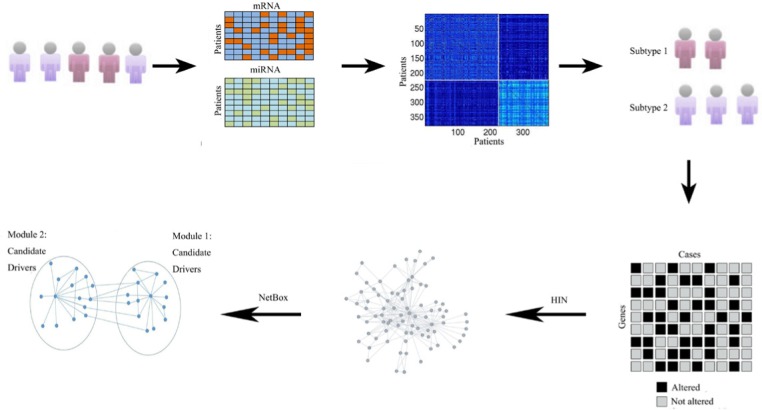
Schematic overview of method used in our study Overview of the approach used for identify core modules for individual subtypes. Given the gene expression and miRNA expression data sets for different patients and genes, the SNF alogrithm fused these two data types and obtained the final cluster. We then extracted the subtype-specific genomic aberration matrix, and utilized a literature curated Human Interaction Network (HIN). Finally, NetBox was used to assess the level of connectivity seen within each of subtype networks and identify the network modules and candidate drivers.

In this study, we developed a novel integrative genomics approach for defining ovarian cancer types and identifying core networks/pathways and driver genes for each subtype. Figure 1 shows the schematic overview of methods used in our study. Firstly, we discovered two molecular subtypes of ovarian cancer by simultaneously clustering mRNA and miRNA expression data derived from TCGA ovarian cancer samples with similarity network fusion (SNF) approach [9]. We then used an integrated network-based approach [6] to identify frequently altered network modules and candidate drivers in each ovarian cancer subtype. Collectively, our result demonstrates the ability of integrative genomics to identify ovarian cancer subtype-specific network modules and candidate drivers.

## RESULTS

### Identification of two molecular subtypes in ovarian cancer

Multiple methods have been applied to identify ovarian cancer subtypes. The use of various data and analysis methods often results in different conclusions. For example, TCGA identified four transcriptional subtypes on the basis of gene expression data [2]. However, these four subtypes show no significant correlation with survival difference. Integrating mRNA and miRNA may be a powerful approach to identify clinically relevant subtypes.

**Figure 2:**
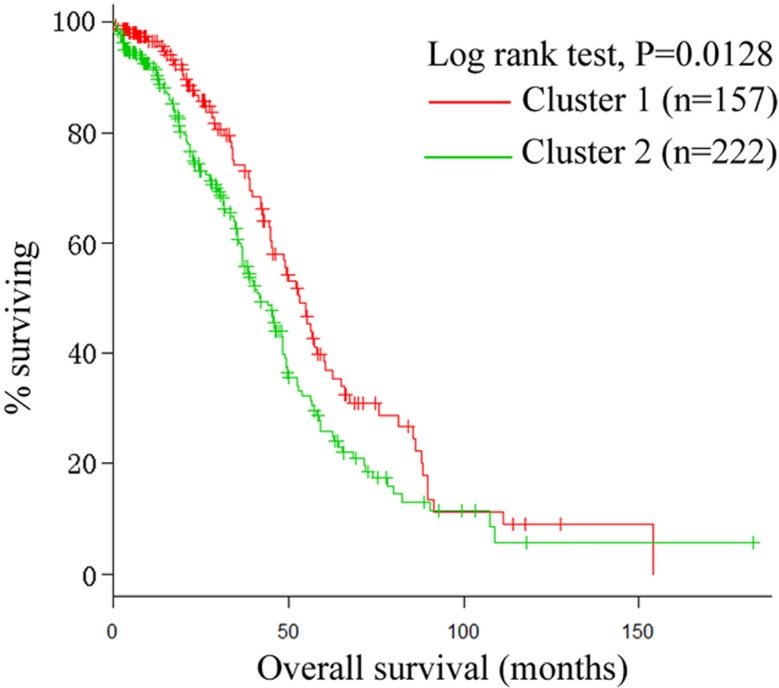
Kaplan-Meier plot The two integrated subtypes of ovarian cancer identified by SNF show survival difference.

In this study, we used SNF [9] to fuse two data types, namely, mRNA expression (17,813 genes) and miRNA expression (798 miRNAs), for 379 ovarian cancer patients. Details are described in the Materials and Methods section. We chose the group with a minimum *P* value in the Cox log-rank test. Figure 2 shows that SNF reliably identified two ovarian cancer subtypes (157 cases in subtype 1 and 222 cases in subtype 2) with distinct survival differences. The majority of patients with subtype 2 ovarian cancer (58.6%; 222 of 379 cases) had significantly shorter overall survival durations than those with subtype 1 ovarian cancer (*P* = 0.0128, log-rank test; Figure 2).

### Properties of the ovarian cancer subtype 1 network

A total of 493 genes that exceeded the frequency threshold were retained and served as altered genes for ovarian cancer subtype 1, as described in the Materials and Methods section. We then used NetBox [6], a well-established method, to extract 56 altered genes and 5 linker genes (linker genes are not altered in ovarian cancer, but are statistically enriched for connections to ovarian cancer altered genes) and identify a total of 8 modules (Supplementary Table S1), with an overall network modularity of 0.326. However, the 1000 simulated random networks have an average modularity of 0.018, with a standard deviation of 0.01. This resulted in a scaled modularity score of 30.8, which indicates that the ovarian cancer subtype 1 network is more modular than random network.

**Figure 3:**
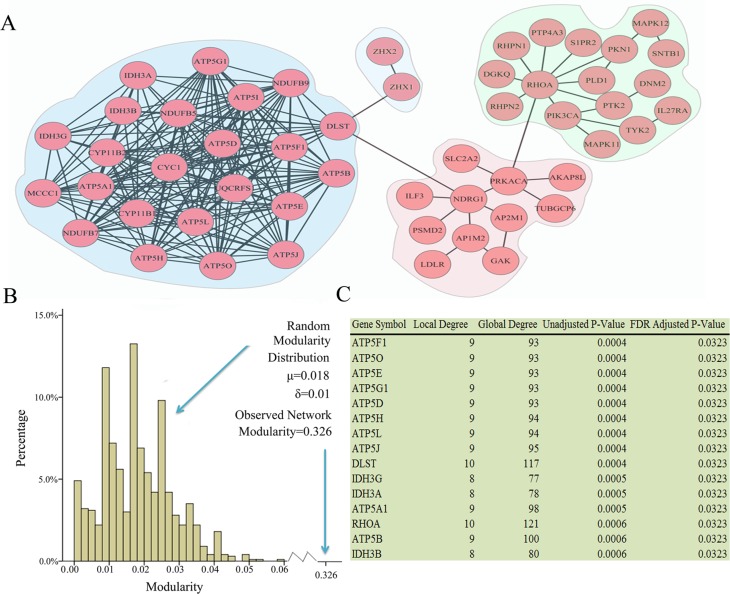
Network modules identified in ovarian cancer subtype 1 (**A**) Modules are closely connected which may reflect oncogenic processes. A total of 8 modules were identified, the largest of which are shown. (**B**) The observed modularity of the ovarian cancer subtype 1 network (0.326) compared with 1000 randomly rewired networks (average 0.018, standard deviation 0.01). (**C**) Linker genes, which are not altered in ovarian subtype 1, but statistically enriched for connections to ovarian cancer subtype1 altered genes.

Among the 8 modules identified in ovarian cancer subtype 1, four are connected and comprise a large network (Figure 3A). These modules are involved in critical signaling pathways. For example, alterations within the RHOA module include *MAPK11* and *MAPK12*, which are members of the p38 MAPK pathway. MAPK signaling is associated with human cancers, including ovarian cancer [10]. Previous study has revealed an association between MAPK expression, and the clinical course of ovarian cancer, which suggests an *in vivo* role for this signal transduction pathway in ovarian carcinoma [11].

We also identified a NDRG1 (N-myc downstream-regulated gene 1) module. *NDRG1* is a cancer-related gene that is strictly up-regulated under hypoxic conditions [12] and is directly targeted by *p53* [13]. Biological experiments have revealed that *NDRG1* was associated with ovarian cancer metastases [14].

**Figure 4:**
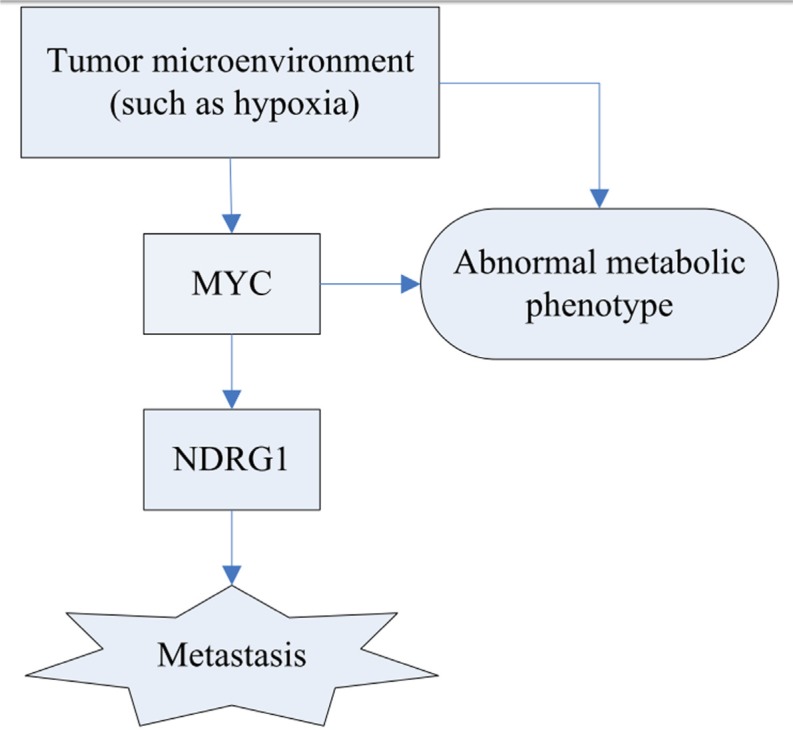
Schematic diagram of the hypoxia regulation and their consequence Tumor metabolism is controlled by intrinsic genetic mutation (*MYC*) and hypoxia. The tumor gene, *MYC*, can regulate the expression of *NDRG1* that potentially improve the likelihood of metastasis.

The most densely interconnected network is the DLST module, which contains many members of metabolic pathways, including those involved in ATP synthase (*ATP5O*, *ATP5D*, *ATP5H*, *ATP5L*, *ATP5G1*, *ATP5J*, *ATP5B*, *ATP5F1*, *ATP5A1*, *ATP5E*, *ATP5I*) [15], and *DLST*, which play a role in the citric acid cycle [16]. As shown in Figure 4, upstream oncogenic pathways that monitor cell conditions can affect metabolism, which leads to the activation of downstream signaling pathways [13]. Bonnet et al. [17] proposed that cancer cells may convert oxidative metabolism to anaerobic metabolism to escape cell death. Overall, accumulating evidence indicated that alterations in metabolic pathways may play a crucial role in ovarian cancer subtype 1 development.

NetBox [6] can also identify candidate driver genes. For example, the most notable and significant candidate gene within the NDRG1 module is *NDRG1*, which connects hypoxic reaction and p53-mediated responses [12]. *ILF3* is another important gene in the NDRG1 module, where it has been shown to be involved in ovarian cancer [18]. *PRKACA* involves in lung cancer epithelial–mesenchymal transition, migration, and invasion [19]. Further evidence suggested that the cAMP signaling pathway can be activated through *PRKACA* mutation in cancer [20].

### Identification of additional modules and candidate drivers for ovarian cancer subtype 1 network

**Figure 5:**
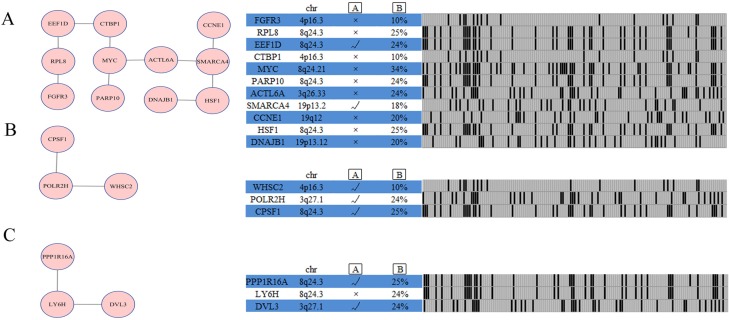
Network analysis identifies additional altered modules for ovarian cancer subtype 1 Each module (Module (**A**): SMARCA4 module; Module (**B**): POLR2H module; Module (**C**): LY6H module) is annotated with chromosome location, statistical significance between copy number and mRNA expression, and genomic status across ovarian cancer subtype one samples. $\begin{array}{c} A \end{array}$ represents gene expression correlates with copy number, as determined by ANOVA analysis across 157 ovarian cancer cases with copy number and expression data. $\begin{array}{c} B \end{array}$ represents percentage of ovarian cancer cases in which gene is altered.

Four additional modules aside from the four main modules were identified by network analysis; three of these modules contain at least three genes (Figure 5).

The SMARCA4 module (Figure 5A) includes 11 genes: *FGFR3*, *RPL8*, *EEF1D*, *CTBP1*, *MYC*, *PARP10*, *ACTL6A*, *SMARCA4*, *CCNE1*, *HSF1*, and *DNAJB1*. *FGFR3* genetic alterations frequently occur in myeloma and bladder cancers, suggesting that this molecule plays a vital role in carcinogenesis [21]. *EEF1D* strongly correlates with gene expression in ovarian clear cell adenocarcinomas [22]. An obvious feature of ovarian cancer is the presence of recurrent regions of copy number gains or losses [2], and rare recurrent genomic events contain known oncogenes [2], such as *MYC* and *CCNE1* in our analysis. The POLR2H module includes three genes, namely, *POLR2H, CPSF1*, and *WHSC2*. Gene expression correlates with the copy number in this module (Figure 5B). The LY6H module is altered in 24% of ovarian cancer subtype 1 cases (Figure 5C) and includes three altered genes, namely, *DVL3*, *LY6H*, and *PPP1R16A*.

### Properties of the ovarian cancer subtype 2 network

A total of 457 genes that exceeded the frequency threshold were retained and served as altered genes for ovarian cancer subtype 2, as described in the Materials and Methods section. After importing these genes into NetBox, 59 altered genes were automatically extracted and five linker genes were identified. Using the module detection algorithm in NetBox, we detected 14 modules (Supplementary Table S2), with an overall network modularity of 0.608. The 1000 simulated random networks have an average modularity of 0.367, with a standard deviation of 0.041. This resulted in a scaled modularity score of 17.2, which indicates that the ovarian cancer subtype 2 network is also more modular than random network.

**Figure 6:**
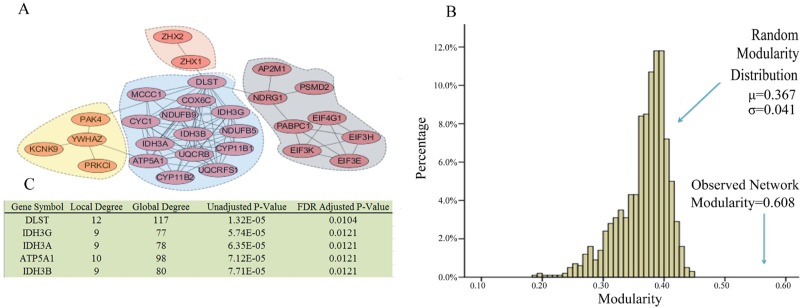
Network modules identified in ovarian cancer subtype 2 (**A**) Modules are closely connected which may reflect oncogenic processes. A total of 14 modules were identified, the largest of which are shown. (**B**) The observed modularity of the ovarian cancer subtype 2 network (0.608) compared with 1000 randomly rewired networks (average 0.367, standard deviation 0.041). (**C**) Linker genes, which are not altered in ovarian subtype two, but are statistically enriched for connections to ovarian cancer subtype 2 altered genes.

The major members of the network modules identified in ovarian cancer subtype 2 are summarized in Figure 6A. The one with the largest density connected in the network is the DLST module. All members of the network are involved in the metabolic pathway, among which *IDH3B*, *IDH3A*, *IDH3G*, and *DLST* participate in the tricarboxylic acid cycle [16, 23, 24]. The relationship between cancer and altered metabolism was observed during the early period of cancer research; it has been demonstrated that altered metabolism is a common phenomenon observed in cancerous tissues [25], which has raised interest in targeting metabolic enzymes of cancer cells [26]. Cancer cells modify their metabolic pathways to satisfy their increasing energy demands during carcinoma proliferation [27]. Cairns [27] considered the alterations of cellular metabolism as a vital hallmark of ovarian cancer. Therefore, drugs that can target cancer metabolism have a great potential in human cancer therapy [26]. For example, *IDH*3A is an up-regulated protein involved in oxidative metabolism in metastatic breast cancer [28]. *IDH3G* was identified in a module and as a hub gene associated with endometrial cancer [29], which is consistent with our results. Therefore, we hypothesized that these genes associated with metabolism contribute to ovarian cancer progression.

The module with the second largest density of internal connections is NDRG1. Figure 6 shows that *NDRG1* is directly connected to *DLST*, suggesting that the NDRG1 module is also involved in metabolism. As previously discussed, the alteration of cellular metabolism is an important feature in cancer. Hypoxia has an effect on tumor metabolism [30]. Hypoxia is an inducer of the *NDRG1* gene that can interact with the oxygen sensory pathway (Figure 4). Therefore, the NDRG1 and DLST modules may combine to regulate the metabolic pathway. In the NDRG1 module, *PSMD2* is overexpressed in many cancer cells [31], whereas *PABPC1*, *EIF4G1*, *EIF3H*, *EIF3E*, and *EIF3K* are RNA translational control members. As a response to tumor stress (e.g., hypoxia), mRNAs encoding proteins are selectively translated because translational control is vital for cancer growth and progression [32].

### Identification of additional modules and candidate drivers for ovarian cancer subtype 2 network

**Figure 7:**
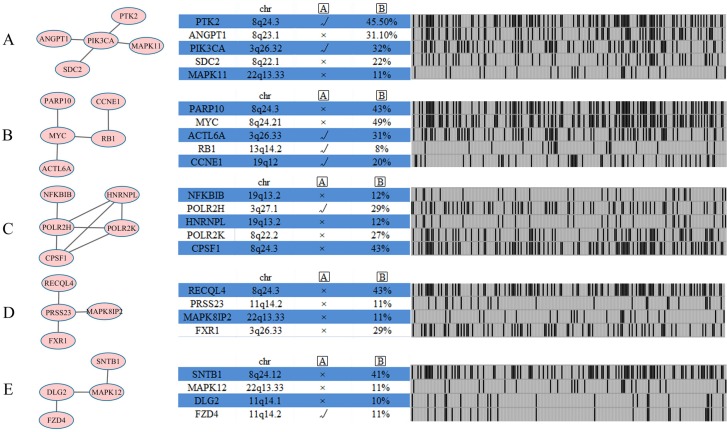
Network analysis identifies five additional altered modules for ovarian cancer subtype 2 Each module (Module (**A**) PIK3CA module; Module (**B**) RB1 module; Module (**C**) CPSF1 module; Module (**D**) PRSS23 module; Module (**E**) MAPK12 module) is annotated with chromosome location, statistical significance between copy number and mRNA expression, and genomic status across ovarian cancer subtype one samples. $\begin{array}{c} A \end{array}$ represents gene expression correlates with copy number, as determined by ANOVA analysis across 222 ovarian cancer cases with copy number and expression data. $\begin{array}{c} B \end{array}$ represents the percentage of ovarian cancer cases in which gene is altered.

We identified several known pathways when searching for the altered networks in HIN (Human Interaction Network) by using NetBox. For example, the RB1 module contains genes *RB1*, MYC, *ACTL6A*, *PARP10*, and *CCNE1* (Figure 7B). Ovarian cancer usually escapes from cell cycle regulation through genetic alterations to the RB pathway [33]. *RB1* is a significantly genetic alteration gene, and the RB pathway is regulated in 67% of ovarian cancer cases [2]. The *RB1* gene is only existed in the module of ovarian cancer subtype 2, and the main difference between the two subtypes is found in the RB pathway. In particular, subtype 2 (with the RB pathway) has a shorter survival rate than subtype 1. Survival analysis among anaplastic astrocytoma cancer samples reveals that dysregulation of the RB signaling pathway negatively correlates with survival [34]. Previous studies have shown the case that cancers with genetic alterations in the RB pathway often have worse overall survival than those without such alterations [35]. Our results were in line with these previous reports [34, 35], suggesting that the two subtype survival differences can be explained by the worse survival of RB pathway subtypes. Aside from *RB1*, *PARP10*, *MYC*, *ACTL6A*, and *CCNE1* are also included in this module. *PARP10* is a MYC-interacting protein that plays a tumor-suppressive role [36]. *MYC* is a key regulator of cell growth and division, deregulation of *MYC* result in uncontrolled cell proliferation and tumor progression [37].

Another PIK3CA module includes five genes (Figure 7A), namely, *PTK2, ANGPT1*, *PIK3CA*, *SDC2*, and *MAPK11*. The CPSF1 module (Figure 7C) also includes five genes: *NFKBIB*, *POLR2H*, *HNRNPL*, *POLR2K*, and *CPSF1*. *NFKBIB* plays a critical role in regulating NF-κB signaling pathway, which involved in key cellular processes, including cell proliferation, cell survival, inflammatory and immune responses [38]. The PRSS23 module (Figure 7D) includes *RECQL4*, *PRSS23*, *MAPK8IP2*, and *FXR1*, and the correlation between gene expression and copy number has no statistical significance in the module. The MAPK12 module (Figure 7E) includes four genes, namely, *SNTB1*, *MAPK12*, *DLG2*, and *FZD4*, among which only *MAPK12* and *DLG2* are associated with kinase activity. The two other modules found are presented in Supplementary Table S2.

### Identification of miRNAs that target RB1

Using the MiRTarbase database [39], we only considered three pieces of evidence with strongly evidence and found 11 miRNAs (miR-132, miR-221, miR-335, miR-192, miR-106a, miR-106b, miR-519a, miR-215, miR-212, miR-26b, miR-26a) that target RB1. We then performed a differential miRNA analysis. Although both of the upregulated and downregulated miRNAs deserve an in-depth investigation, here we focused on the upregulated miRNAs that tend to reduce RB1 expression. *P*-value < 0.01 and fold change > 1.2 were defined as upregulated miRNA, and miR-132 (*P*-value: 2.6 × 10^−7^, fold change: 1.2), miR-221(*P*-value: 4 × 10^−4^, fold change: 1.3), and miR-212(*P*-value: 1.3 × 10^−7^, fold change: 1.2) were obtained.

Both miR-132 and miR-212 are located on chromosome 17p13, which were predicted to target the tumor suppressor *RB1* and reduce its levels. miR-132/-212 is reportedly overexpressed in pancreatic cancer [40], but the cause of miR-132/-212 upregulation in ovarian cancer subtype remains unknown. We proposed that miR-132/-212 targets RB1 and promotes tumor proliferation. Several studies have indicated that high expression levels of miR-221 in tumor tissues are associated with overall survival in hepatocellular carcinoma, T-cell acute lymphoid leukemia, thyroid papillary carcinoma, pancreatic adenocarcinoma, and GBM [41, 42]. Hong et al. [43] reported that high serum miR-221 expression in human epithelial ovarian cancer correlates with short overall survival. Therefore, miR-221 may play an important role in the progression of malignancies. The molecular mechanism that links miR-221 overexpression to short overall survival is not well understood. In addition, a one-to-one correspondence between miRNAs and their target genes does not exist. Therefore, identifying the miRNAs that are important for regulating RB1 remains a crucial aspect of future investigations.

### Pathways associated with ovarian cancer subtype-specific network

We conducted pathway analysis for each ovarian cancer subtype using an ontology-based pathway database [44] in DAVID [45] to associate ovarian cancer subtype-specific networks with known pathways (Supplementary Table S3). We filtered out GO terms with an adjusted *P* > 0.05. Angiogenesis and p53 pathway were enriched in two ovarian cancer subtypes, although at different levels of significance. It is notably that angiogenesis is a hallmark of cancer [46]. In addition, the ovarian cancer subtype 1 were markedly enriched with genes in the Ras Pathway, and subtypes 2 was enriched with genes in VEGF signaling pathway and B cell activation. Previous study has revealed that the enhanced expression of VEGF are correlated with patient survival and tumor metastasis [47]. These results suggest that targeting the VEGF pathway or simultaneously targeting the VEGF and B cell activation pathway seem like rational choices for ovarian cancer subtype 2 patients, which have a shorter survival time compared with ovarian cancer subtype 1 patients.

## DISCUSSION

Previous studies usually stimulated one data type to identify ovarian cancer subtypes, whereas we systematically identified two subtypes in ovarian cancer by integrating mRNA and miRNA data. Compared to other three ovarian cancer subtypes (log-rank test, *P* = 0.043) identified by Yuan et al. [8], our identified two subtypes have a significant overall survival difference (log-rank test, *P* = 0.0128) between the two subtypes.

After discovering two ovarian cancer subtypes, we used a subtype-based core module inference strategy to decipher subtype genomic alterations. Instead, existing studies often focus on network analysis in cancer cohorts without considering cancer subtypes. These methods inclined to identify networks common in many neoplasm samples and ignore the heterogeneity among cancer subtypes.

We explored the CNA (copy number alternation) among the two subtypes in ovarian cancer and identified core modules that provide a consistent and integrated picture of two subtypes and link genomic alternations to biological processes. Our analysis reveals that genomic alterations in the metabolism and NDRGD1 pathway were common in the two subtypes, consistent with its critical role in the initiation of each subtype. However, the cohesion of core module networks drives distinct molecular phenotypes in the two ovarian cancer subtypes. Activation of both NDRGD1 and DLST modules may promote the metabolic signaling pathway. For subtype 2, genomic alterations in the RB signaling pathway may activate the pathway continually and disrupt cell cycle and proliferation [48]. Moreover, the RB pathway has a strong association with poor survival in many cancers, including GBM [35] and ovarian cancer [49]. Therefore, alternations in the RB signaling pathway may prompt subtype 2 to suffer a poor clinical outcome (Table 1). On the contrary, no RB1 signaling pathway may help subtype 1 result in a favorable clinical outcome.

**Table 1:** Distinct core modules driving two ovarian cancer subtypes biology and clinical outcome

		Main module			5yr-survival rate	
	Metabolism	NDRG1	RB1	ZHX1	Stage I–IV	Stage III–IV
Subtype 1	√	√	×	√	16.7%	15.4%
Subtype 2	√	√	√	√	12.7%	11.7%

Our findings not only present a comprehensive understanding of ovarian cancer but also provide guidance orientation on possible personalized therapeutic approaches for different subtypes. Targeting the metabolic pathway or simultaneously targeting the RB signaling pathway is a rational choice for efficient cancer therapy. However, clinical trials on targeting metabolic pathway and RB pathway therapies for ovarian cancer patients have reached inconsistent conclusions [26, 50]. Therefore, clinical trials should consider the cancer subtypes of patients because subtype 2 (with activated RB signaling pathway) may be more sensitive to anti-RB therapy than subtype 1.

In summary, our study elucidated the molecular mechanisms underlying ovarian cancer subtypes and helped identify candidate driver genes by using an automated network method. Heterogeneity is a common phenomenon in cancer, and our results implied that heterogeneity also exists in the two subtypes. In the future, individualized treatment should not be confined to the therapeutic strategies for ovarian subtype-specific therapy; rather, patient-specific driver gene predictions and therapy should be the focus of medical development. Potential driver modules may serve as determining factors for cancer progression and survival time. Further studies are required to explore this hypothesis.

## MATERIALS AND METHODS

### Data acquisition and processing

We downloaded gene expression (Agilent G4502A) data, miRNA (Agilent 8 × 15 K human miRNA-specific microarray platforms) data, survival data, and clinical data from the Synapse website (http://www.synapse.org, accession number syn1710282). DNA copy number data were obtained from the TCGA (https://tcga-data.nci.nih.gov/tcga/), which was analyzed by GISTIC2.0 [51]. Only the homozygous deletions and amplifications were considered as copy number alternations (CNAs). We generated a binary matrix of genetic alteration, in which score 1 represents the *i*th gene in the *j*th sample with a genetic alteration, and score 0 represents otherwise. Some samples contained several genetic alteration genes, whereas other samples contained numerous genetic alteration genes. To assign a higher weight to this genomic alteration, we considered column-wise normalization, which mainly contains two steps. First, we performed row-wise summarization for each matrix; second, we converted each matrix into a vector. For each subtype, *n* denotes the total number of genes in each group and *m* denotes the number of each subtype sample. The score of gene *i* in genetic alteration matrix *C* is defined as

$$C_{i}=\sum_{j=1}^{m}\frac{x_{ij}}{\sum_{i=1}x_{ij}}.$$ (1)

where X_ij_ corresponds to gene *i* in sample *j* in the binary matrices. All C_i_ for each gene were given equal weight. The probability for gene *i* (*p*_i_^0^) was computed as

$$\rho_{i}^{0}=\frac{C_{i}}{\sum_{i=1}^{n}C_{i}}.$$ (2)

On the basis of the genetic alteration matrix with 17,814 genes and 157 samples for subtype 1 and 17,814 genes and 222 samples for subtype 2, we calculated gene (*p*_i_^0^) in subtypes 1 and 2. Gene scores greater than or equal to 2 × 10^−4^ were considered. After filtration, 458 and 666 altered genes were selected for subtypes 1 and 2, respectively. Then these altered genes were imported into NetBox and used for module detection.

### Subtype identification

We applied the SNF [9] algorithm for a joint analysis of gene mRNA expression (12,042 genes) and miRNA expression (534 miRNAs) on a subset of 379 ovarian cancers. The main procedure of the subtype identification contains two parts.

### Similarity network fusion

We constructed a graph *G* = (*V, E*) that represents a patient similarity network for each of the two available datasets. The vertices *V* correspond to the patients, whereas the edges *E* are weighted by the similarity between the patients. A weight matrix *W* represents all edges, with *W(i, j)* indicating the similarity between patients *i* and *j*. The weight matrix *W* was defined as:

$$W(i,j)=\text{exp}\left(-\frac{\rho^{2}X_{j},X_{j}}{\mu \varepsilon_{i,j}}\right),$$ (3)

where *μ* is a hyperparameter that can be empirically set, *ρ*(*x_i_*, *x_j_*) is the Euclidean distance between *i* and *j*, and ε_i, j_ is the means to eliminate the scaling problem. Here, we defined ε_i, j_ as

$$\varepsilon_{i,j}=\frac{\text{mean}(\rho (x_{i},N_{i}))+\text{mean}(\rho (x_{j},N_{j}))+\rho (x_{i},x_{j})}{3},$$ (4)

where mean (*ρ*(*X_i_*, *N_i_*) is the average value of the distance between x_i_ and its neighbors.

After defining a weight matrix, a normalized weight matrix *P* was acquired as follows:

$$P(i,j)=\{\begin{array}{c} \frac{W(i,j)}{2\sum_{k\neq i}W(i,k)},j\neq i\\ 1/2,\;j=i \end{array}.$$ (5)

The normalization is free of the scale of self-similarity in the diagonal entries and avoids numerical instability.

To measure the local affinity of a node *i* to all its neighbors *N_i_*, k-nearest neighbors method was used:

$$S\left(i,j\right)=\{\begin{array}{c} \frac{W\left(i,j\right)}{\sum_{\kappa \in N_{i}}W\left(i,k\right)},j\in N_{i}\\ 0\;,\text{ otherwise} \end{array}.$$ (6)

*S* only retained the k-nearest neighbors for each patient and filtered out low-similarity edges.

The similarity matrices *P*^(*v)*^ and *S*^(*v)*^ were calculated from the dataset v. SNF iterated each dataset's similarity matrix and was defined as

$$P^{\left(i\right)}=S^{\left(v\right)}\times \left(\frac{\sum_{k\neq v}P^{\left(k\right)}}{m-1}\right)\times {\left(S^{\left(v\right)}\right)}^{T},v=1,\;2,\;\ldots ,m.$$ (7)

This procedure updates the matrices *P* each time, meanwhile, it generates *m* parallel interchanging diffusion procession in *m* networks. If vertices *i* and *j* are similar in all of the data types, then their similarity will be improved through diffusion and vice versa.

### Spectral clustering (ovarian cancer subtyping)

To identify *C* cluster samples (each cluster represents a subtype), we defined a label indicator vector y_*i.*_ If patient *i* belongs to the *k*th subtype, we define the *y_i_* (k) = 0; otherwise, *y_i_* (k) = 1. Thus a partition matrix Y = (*y*_1_^T^, *y*_2_^T^,…*y_n_*^T^) delineates a clustering scheme.

We clustered the patient samples in the fused similarity matrix L^+^ = I–D^−1/2^ WD^−1/2^ by using spectral clustering. The normalized Laplacian matrix was with the final similarity matrix P, and the network degree matrix D was with the scaled partition matrix Q = Y(Y^T^Y)^−1/2^. The spectral clustering plans to minimize the objective function were as follows:

$${\text{min}}_{Q\in R}n\times c\text{ Trace}(Q^{T}L^{+}Q)s.t.Q^{T}Q=I$$ (8)

### Identification of core modules in ovarian cancer subtypes

The construction of subtype-specific network modules in NetBox [6] allows us to explore the functional relevance of genes in a defined network. NetBox was used to explore the subtype-specific modules in a defined literature curated human interaction network. Once the altered gene in each of subtypes which altered by copy number alternation were inputted into Netbox, module detection is automatically. (NetBox was used to explore the subtype-specific modules in a defined literature curated human interaction network.) Genes in the detected modules have a potential to be driver genes. 458 and 666 altered genes were imported into NetBox, respectively. Using the global null model and local null module in NetBox, the level of connectivity seen within the subtype-specific modules were assessed.

NetBox identifies the intermediate genes (linker genes) for the connection of input genes through subnetwork extraction. Before running NetBox, we set the neighbor nodes of degree to 2, allowing the linker gene to connect two altered genes within the network. To identify significant linker genes in statistics, we adjusted the *p*-value using Benjamini–Hochberg [52] and set the threshold at 0.035 for subtype 1 and 0.0125 for subtype 2. We ran 1000 iterations to evaluate the level of global network connectivity in the ovarian cancer subtypes. We randomly selected the same number of altered genes in each iteration and connected them via the original shortest path threshold and *p*-value cut-off parameters.

### Network visualization and module analysis

Networks were visualized in Cytoscape [53], and modules were visualized across the two subtypes in ovarian cancer. The correlation coefficient between CNA and mRNA expression was calculated via analysis of variance in R version 2.7.2.

### Statistical analysis

Statistical calculation was performed in R version 2.7.2. To analyze the survival of patients, log rank *p*-values were computed using the R package “survival.” The Wilcoxon–Wilcox test was used to analyze the miRNAs between the two subtypes.

### GO analysis

We performed the functional analysis on DAVID [45] online service to evaluate the function of each driver gene set in subtypes.

## SUPPLEMENTARY MATERIAL TABLES


