# Supplementary material for: Identification of ovarian cancer subtype-specific network modules and candidate drivers through an integrative genomics approach
Source: Oncotarget. 2015 Dec 28;7(4):4298–309. doi: 10.18632/oncotarget.6774 (PMC4826206; doi:10.18632/oncotarget.6774)
Supplement: Supplementary file 1 [file oncotarget-07-4298-s001.pdf]

Identification of ovarian cancer subtype-specific network modules and candidate drivers through an integrative genomics approach

Supplementary Materials

Supplementary Table S1: 8 modules identified in ovarian cancer subtype 1 network

Module label	Number of genes	Genes
RHOA	16	RHPN1(1) RHPN2(1) SNTB1(1) RHOA*(10) DNM2(1) PLD1(2) DGKQ(1) PKN1(3) MAPK12(2) PTK2(3) IL27RA(1) MAPK11(1) TYK2(2) S1PR2(1) PTP4A3(1) PIK3CA(4)
SMARCA4	11	DNAJB1(1) FGFR3(1) RPL8(2) HSF1(2) CTBP1(2) CCNE1(1) MYC(3) ACTL6A(2) EEF1D(2) PARP10(1) SMARCA4(3)
NDRG1	11	AKAP8L(1) GAK(1) TUBGCP6(1) AP1M2(3) ILF3(1) SLC2A2(1) AP2M1(2) PRKACA(5) NDRG1(6) PSMD2(1) LDLR(1)
DLST	23	ATP5O*(18) ATP5D*(18) ATP5H*(18) ATP5L*(18) IDH3G*(11) NDUFB5(19) ATP5G1*(18) NDUFB9(19) IDH3B*(11) ATP5J*(18) DLST*(13) ATP5B*(18) CYC1(19) ATP5F1*(18) IDH3A*(11) CYP11B2(16) MCCC1(15) ATP5A1*(18) UQCRFS1(19) ATP5E*(18) NDUFB7(19) CYP11B1(16) ATP5I(18)
POLR2H	3	CPSF1(1) POLR2H(2) WHSC2(1)
ABCF3	2	ABCF3(1) SLC35E1(1)
LY6H	3	LY6H(2) DVL3(1) PPP1R16A(1)
ZHX1	2	ZHX1(2) ZHX2(1)

*Linker gene is not altered in ovarian cancer, but is statistically enriched for connections to ovarian cancer altered genes. The number in brackets is the degree of gene.

Supplementary Table S2: 14 modules identified in ovarian cancer subtype 2 network

Module label	Number of genes	Genes
PDX1	3	SLC2A2(1) MAFA(1) PDX1(2)
ZHX1	2	ZHX2(1) ZHX1(2)
DLST	14	IDH3B*(12) CYC1(10) MCCC1(5) IDH3A*(12) CYP11B2(6) COX6C(8) NDUFB5(9) CYP11B1(6) NDUFB9(9) UQCRFS1(10) ATP5A1*(10) IDH3G*(12) DLST*(15) UQCRB(10)
RB1	5	MYC(3) ACTL6A(1) RB1(2) PARP10(1) CCNE1(1)
EEF1D	3	EEF1D(2) RPL8(2) RPL30(2)
CPSF1	5	NFKBIB(1) HNRNPL(3) CPSF1(3) POLR2K(3) POLR2H(4)
MAPK12	4	DLG2(2) SNTB1(1) MAPK12(2) FZD4(1)
PRSS23	4	PRSS23(3) FXR1(1) RECQ L4(1) MAPK8IP2(1)
TNFSF10	2	TNFSF10(1) TNFRSF11B(1)
NDRG1	8	EIF3H(4) PABPC1(5) AP2M1(1) EIF3K(4) PSMD2(1) EIF4G1(4) EIF3E(4) NDRG1(4)
PIK3CA	5	SDC2(1) MAPK11(1) PTK2(1) PIK3CA(4) ANGPT1(1)
LY6H	3	DVL3(1) PPP1R16A(1) LY6H(2)
POP1	2	POP4(1) POP1(1)
YWHAZ	4	KCNK9(1) PAK4(2) YWHAZ(4) PRKCI(1)

*Linker gene is not altered in ovarian cancer, but is statistically enriched for connections to ovarian cancer altered genes. The number in brackets is the degree of gene.

Supplementary Table S3: Pathways associated with ovarian cancer subtype

(A) Subtype 1

Annotation source	Term	Count	Raw *P*-value	Benjamini-Hochberg adjusted *P*-value
PANTHER_PATHWAY	P00005:Angiogenesis	8	0.000349139	0.01902272
PANTHER_PATHWAY	P04398:p53 pathway feedback loops 2	5	0.000610762	0.016660739
PANTHER_PATHWAY	P04393:Ras Pathway	5	0.002483911	0.044571194

Note: Count represents the number of annotated genes in each pathway term.

(B) Subtype 2

Annotation source	Term	Count	Raw *P*-value	Benjamini-Hochberg adjusted *P*-value
PANTHER_PATHWAY	P04398:p53 pathway feedback loops 2	6	0.0000376	0.002068051
PANTHER_PATHWAY	P00005:Angiogenesis	8	0.000349	0.009557028
PANTHER_PATHWAY	P00056:VEGF signaling pathway	5	0.001459193	0.026416224
PANTHER_PATHWAY	P00010:B cell activation	5	0.002010594	0.02729409

Note: Count represents the number of annotated genes in each pathway term.
